# Generalizations of the short pulse equation

**DOI:** 10.1007/s11005-017-1022-3

**Published:** 2017-11-10

**Authors:** Andrew N. W. Hone, Vladimir Novikov, Jing Ping Wang

**Affiliations:** 1 School of Mathematics, Statistics and Actuarial Science, University of Kent, Canterbury, CT2 7FS UK; 2 Department of Mathematical Sciences, Loughborough University, Loughborough, LE11 3TU UK

**Keywords:** Short-wave limit, Symmetries, Recursion operator, Lax pair, 37K10, 35G20

## Abstract

We classify integrable scalar polynomial partial differential equations of second order generalizing the short pulse equation.

## Introduction

The purpose of this short article is to present a classification of nonlinear partial differential equations of second order of the general form

1 $$\begin{aligned} u_{xt}=u+c_0u^2+c_1uu_x+c_2uu_{xx}+c_3u_x^2+d_0u^3+d_1u^2u_x+d_2u^2u_{xx}+d_3uu_x^2, \end{aligned}$$

which in the case that $c_j=0$ for $j=0,1,2,3$ and $d_0=d_1=0$, $d_3=2d_2$, includes the short pulse equation derived by Schäfer and Wayne [29] as a model of ultra-short optical pulses in nonlinear media; cf. Eq. (3) below. It was shown by Sakovich and Sakovich [27] that the short pulse equation is integrable, in the sense that it admits a Lax pair and a recursion operator that generates infinitely many commuting symmetries; these authors also found a hodograph-type transformation connecting it with the sine-Gordon equation. In fact, the short pulse equation and the construction of its associated linear scattering problem first appeared in differential geometry [1, 26]. The integrability of the equation was further clarified by Brunelli [2], who obtained a bi-Hamiltonian structure and used an alternative Lax representation to construct an infinite sequence of conserved quantities.

Short pulses and their properties are a subject of current interest in nonlinear optics and electrodynamics, both theoretically and experimentally. For instance, a rigorous justification of the short pulse equation, starting from a quasilinear Klein-Gordon equation (a toy model for Maxwell’s equations) was given in [25]. Moreover, for electrons accelerated in short laser pulses, it was shown recently that, due to quantum effects, the radiation reaction can be quenched by suitably tuning the pulse length, although the lengths required are currently out of experimental reach [15].

In this paper we are concerned with generalized short pulse equations of the form (1) from the viewpoint of integrability. The main result of the paper is the following.

### Theorem

If the Eq. (1) possesses an infinite hierarchy of local higher symmetries, then up to rescaling $u\rightarrow {\lambda }u,\,x\rightarrow \mu x,\, t\rightarrow \nu t$ it is one of the list

2 $$\begin{aligned} u_{xt}= & {} u+ \left( u^2\right) _{xx}, \end{aligned}$$

3 $$\begin{aligned} u_{xt}= & {} u+\left( u^3\right) _{xx},\end{aligned}$$

4 $$\begin{aligned} u_{xt}= & {} u+4uu_{xx}+u_x^2, \end{aligned}$$

5 $$\begin{aligned} u_{xt}= & {} u+\left( u^2-4u^2u_x\right) _{x},\end{aligned}$$

6 $$\begin{aligned} u_{xt}= & {} u+2uu_{xx}+u_x^2,\end{aligned}$$

7 $$\begin{aligned} u_{xt}= & {} u+u^2u_{xx}+uu_x^2,\end{aligned}$$

8 $$\begin{aligned} u_{xt}= & {} u+\alpha \left( 2uu_{xx}+u_x^2\right) +\beta \left( u^2u_{xx}+uu_x^2\right) , \qquad {\alpha }{\beta }\ne 0 . \end{aligned}$$

### Remark

The nonlinear terms in Eq. (8) are a linear combination of those in Eqs. (6) and (7). Upon applying an affine linear transformation $u\rightarrow au+b$ together with a Galilean transformation $x\rightarrow x-ct$ for suitable *a*, *b*, *c*, the derivative of (8), that is

$$\begin{aligned} u_{xxt}= u_x+\alpha \Big (2uu_{xx}+u_x^2\Big )_x+\beta \Big ( u^2u_{xx}+uu_x^2\Big )_x, \end{aligned}$$

can be transformed to the case ${\alpha }=0$, ${\beta }=1$, which is the derivative of (7); but for the original Eq. (8) the quadratic terms cannot be removed in this way.

Equations of the form (1) are of interest for various reasons. Observe that, as written, (1) is not an evolution equation for *u*, and if it is rewritten as one, solving for $u_t$, then it becomes nonlocal, involving the integration operator $D_x^{-1}$. Physically, such equations appear in the description of the short-wave behavior of nonlinear systems. For example, the *b*-family of equations

9 $$\begin{aligned} m_t+um_x+bu_xm=0, \qquad m=m_0 +u-u_{xx} , \qquad m_0={\mathrm {const}}, \end{aligned}$$

which was introduced in [8] (see also [9, 19]) and derived from shallow water theory in [6, 10], has a short-wave limit found by setting

$$\begin{aligned} x\rightarrow \epsilon x, \qquad t\rightarrow \epsilon t, \qquad m_0\rightarrow -\mu \epsilon ^{-2},\qquad \mu ={\mathrm {const}}, \end{aligned}$$

and then taking $\epsilon \rightarrow 0$, which yields

10 $$\begin{aligned} u_{xxt}+b\mu u_x + uu_{xxx}+bu_xu_{xx}=\Big (u_{xt}+b\mu u + uu_{xx}+\frac{1}{2}(b-1)u_x^2\Big )_x = 0.\nonumber \\ \end{aligned}$$

After sending $t\rightarrow -t$ and rescaling *u*, (10) is seen to be the *x* derivative of an equation of the form (1). It is known from [22] that (for $b\ne 0$) the Eq. (9) is integrable, in the sense that it admits an infinite hierarchy of commuting symmetries, if and only if $b=2$ (Camassa–Holm [3]) or $b=3$ (Degasperis–Procesi [7]). Surprisingly, comparison with (2), (4) and (6) in the above theorem shows that in the short-wave limit, there are three integrable cases of Eq. (10): not only $b=2$ (Hunter–Saxton [17]) and $b=3$ (Vakhnenko [30]), but also the case $b=3/2$, which appears to be new.

The proof of the above theorem consists of two parts. The first part consists of applying the perturbative symmetry approach, as described in [22], to obtain a set of necessary conditions on the parameters $c_j,d_j$ in (1) for the existence of a formal recursion operator with local coefficients (i.e., functions of *u* and its derivatives only). This part of the proof requires the use of computer algebra, and further details are omitted. Once a finite list of equations has been obtained as above (by scaling $c_j,d_j$ suitably), the remainder of the proof consists of explicitly constructing a recursion operator and associated infinite hierarchy of symmetries for each equation found. Thus, in the rest of the paper, we consider each equation on the list in turn, and for each one present the first higher symmetry, with flow variable $\tau$, and a recursion operator $\mathcal R$. The recursion operator is factored as $\mathcal{R}=\mathcal{H}\mathcal{J}$, in terms of a compatible implectic–symplectic pair, with $\mathcal{H}$ being a Hamiltonian operator such that the flow can be written as

$$\begin{aligned} u_\tau =\mathcal{H}\, \delta _u \, \rho , \end{aligned}$$

where $\rho$ is a density and $\delta _u$ denotes the variational derivative, i.e.,

$$\begin{aligned} \delta _u \, \rho =\frac{\delta H}{\delta u} , \qquad {\mathrm {where}} \quad H=\int \rho \,\mathrm {d}x \end{aligned}$$

is the Hamiltonian functional with density $\rho$; and the flow is also written as

$$\begin{aligned} \mathcal{J}\, u_\tau = \delta _u \, \tilde{\rho }, \end{aligned}$$

using the symplectic operator $\mathcal{J}$ with another density $\tilde{\rho }$. In addition, for each item on the list, we use a conservation law to define a reciprocal transformation, i.e., a change of independent variables of hodograph type, which provides a link to other known integrable equations. We also present a Lax pair in each case.

Throughout the paper, subscripts with numbers are used to denote higher derivatives, so that $u_{nx}=\frac{\partial ^n u}{\partial x^n}$ for $n\ge 2$, but we also write, e.g., $u_{xx}=u_{2x}$.

## Properties of the generalized short pulse equations

### Vakhnenko’s equation

The Eq. (2) was derived by Vakhnenko [30] as a model for the propagation of short-wave perturbations in a relaxing medium. Its loop soliton solutions were studied extensively in [23, 31]. In [18], it was shown that the *x* derivative of (2) arises as a short-wave, high-frequency limit of the Degasperis–Procesi equation. Sometimes (2) is also referred to as the reduced Ostrovsky equation [12, 13], since (up to rescaling dependent and independent variables) it is the special case ${\beta }= 0$ of the Ostrovksy equation

$$\begin{aligned} \Big ( u_t + uu_x + {\beta }u_{xxx}\Big )_x -{\gamma }u =0, \end{aligned}$$

which is a model of weakly nonlinear ocean waves under the influence of the Coriolis force [24].

*Higher symmetry* The first higher symmetry of the Eq. (2) is

11 $$\begin{aligned} u_{\tau }=\left( \frac{u_{3x}}{(1+6u_{2x})^{\frac{5}{3}}}\right) _{xx}. \end{aligned}$$

*Hamiltonian structure and recursion operator* In terms of the quantity

$$\begin{aligned} w=(6 u_{xx}+1)^{-\frac{1}{3}}, \end{aligned}$$

the symmetry (11) becomes

12 $$\begin{aligned} w_{\tau }=w^5w_{5x}+5 w^4 w_x w_{4x}+10 w^4 w_{2x}w_{3x} \end{aligned}$$

which takes the form

$$\begin{aligned} w_{\tau }=-\frac{1}{2} \mathcal{H}\delta _w w^{-1} , \qquad {\mathrm {where}} \quad \mathcal{H}=w^4 D_x^5 w^4 \end{aligned}$$

is a Hamiltonian operator. The associated symplectic operator is

13 $$\begin{aligned} \mathcal{J}= & {} w^{-2} D_x+D_x w^{-2} +\left( 2 w^{-1} w_{2x}-w^{-2} w_x^2\right) D_x^{-1} w^{-2}\nonumber \\&+\,w^{-2} D_x^{-1} \left( 2 w^{-1} w_{2x}-w^{-2} w_x^2\right) . \end{aligned}$$

Thus, the recursion operator $\mathcal{R}=\mathcal{H}\mathcal{J}$ generates the symmetries for (2) and

$$\begin{aligned} \mathcal{J}w_{\tau }=\delta _w \left( -w^3 w_{3x}^2+\frac{8}{3} w^2 w_{2x}^3-4 w w_x^2 w_{2x}^2-\frac{w_x^6}{3 w} \right) . \end{aligned}$$

*Reciprocal transformation* Viewed as a short-wave limit of the Degasperis–Procesi equation, the *x* derivative of (2) can be written in the form

$$\begin{aligned} m_t =2um_x+6u_xm, \qquad m=1+6u_{xx} , \end{aligned}$$

giving a conservation law for the density $p=w^{-1}=(1+6u_{xx})^{1/3}$, that is

14 $$\begin{aligned} p_t=2\, (up)_x, \qquad p^3=m. \end{aligned}$$

This conservation law leads to the introduction of new independent variables *X*, *T* by means of the reciprocal transformation

$$\begin{aligned} \mathrm {d}X=p \, \mathrm {d}x+2pu\, \mathrm {d}t, \qquad \mathrm {d}T= \mathrm {d}t, \end{aligned}$$

so that (14) produces

15 $$\begin{aligned} \left( p^{-1}\right) _T +2 u_X=0, \qquad p^3= 1+6p(pu_X)_X. \end{aligned}$$

If we use the letter *W* to denote $u_x$, then we have

$$\begin{aligned} W=pu_X. \end{aligned}$$

The Eq. (2) becomes

$$\begin{aligned} W_T=u+2W^2, \end{aligned}$$

and (15) can be rewritten as the pair of relations

$$\begin{aligned} (\log p)_T = W, \qquad p^3-1 = 6pW_X, \end{aligned}$$

which implies that *p* satisfies the Tzitzeica equation in the form

$$\begin{aligned} (\log p)_{XT} = \frac{1}{6}\Big ( p^2 - p^{-1}\Big ). \end{aligned}$$

*Lax pair* In [32], a scalar Lax pair was presented for a reciprocally transformed version of (2), and in [18] this was used to obtain a $3\times 3$ matrix Lax pair for the original equation, which is equivalent to the following Lax representation with spectral parameter ${\lambda }$:

16 $$\begin{aligned} {\varvec{\Phi }}_x=\left( \begin{array}{ccc} 0 &{} 1 &{} 0 \\ -2\lambda u_x &{} 0 &{} 1\\ -\frac{1}{3}\lambda &{} 2\lambda u_x &{} 0 \end{array}\right) \, {\varvec{\Phi }}, \quad {\varvec{\Phi }}_t=\left( \begin{array}{ccc} 0&{} 2u &{} -{\lambda }^{-1} \\ \frac{1}{3}(1-12\lambda u u_x)&{} 0 &{} 2u\\ \frac{4}{3}{\lambda }u&{} \frac{1}{3}(1+12\lambda u u_x)&{}0 \end{array}\right) \, {\varvec{\Phi }}.\nonumber \\ \end{aligned}$$

### The short pulse equation

The short pulse equation was first derived as an equation for pseudospherical surfaces with an associated inverse scattering problem [1, 26]. Its physical derivation in nonlinear optics came later [29] and led to the construction of alternative forms of the Lax pair, recursion operator and bi-Hamiltonian structure [2, 27].

*Higher symmetry* The first higher symmetry of the Eq. (3) is

$$\begin{aligned} u_{\tau }= \left( \frac{u_x}{\left( 1+6u_x^2\right) ^{\frac{1}{2}}}\right) _{xx}. \end{aligned}$$

*Hamiltonian structure and recursion operator* The above symmetry takes the Hamiltonian form

$$\begin{aligned} u_{\tau } =-\frac{1}{6}D_x \delta _u p \qquad p= \left( 6 u_x^2+1\right) ^{\frac{1}{2}}, \end{aligned}$$

with $\mathcal{H}=D_x$ being the Hamiltonian operator. The symmetries of Eq. (3) are generated by the recursion operator

$$\begin{aligned} \mathcal{R}= D_x\mathcal{J}= D_x \left( p^{-1} D_x p^{-1} +6 p^{-3} u_{2x} D_x^{-1} p^{-3} u_{2x} \right) , \end{aligned}$$

and

$$\begin{aligned} \mathcal{J}u_{\tau }= \delta _u \left( \frac{p^{-5} u_{2x}^2}{2}\right) . \end{aligned}$$

*Reciprocal transformation* The Eq. (3) has the conservation law

17 $$\begin{aligned} p_t=3\, \left( u^2 p\right) _x, \end{aligned}$$

which leads to the introduction of new independent variables *X*, *T* according to

$$\begin{aligned} \mathrm {d}X= p\, \mathrm {d}x+ 3u^2 p \, \mathrm {d}t, \qquad \mathrm {d}T=\mathrm {d}t. \end{aligned}$$

In the new variables, the conservation law becomes

$$\begin{aligned} \left( p^{-1}\right) _T+3\,\left( u^2\right) _X=0, \end{aligned}$$

and by setting $W=u_x$ the original Eq. (3) gives

18 $$\begin{aligned} W_T=up^2, \end{aligned}$$

where we have used

19 $$\begin{aligned} p^2 = 1+6W^2, \qquad u_X=\frac{W}{p}. \end{aligned}$$

Now if a new dependent variable is introduced as

$$\begin{aligned} \varphi = -\frac{\mathrm {i}}{2}\log \left( \frac{1+\mathrm {i}\sqrt{6}W}{1-\mathrm {i}\sqrt{6}W}\right) , \end{aligned}$$

then from (18) and (19), it follows that

$$\begin{aligned} u=\frac{\varphi _T}{\sqrt{6}}, \end{aligned}$$

and $\varphi$ satisfies the sine-Gordon equation, that is

$$\begin{aligned} \varphi _{XT}=\sin \varphi . \end{aligned}$$

*Lax pair* Equation (3) admits the Lax representation

20 $$\begin{aligned} {\varvec{\Phi }}_x= & {} \left( \begin{array}{cc} 0 &{} 1+\mathrm {i}\sqrt{6}u_{x} \\ -\lambda (1-\mathrm {i}\sqrt{6}u_x) &{} 0 \end{array}\right) \, {\varvec{\Phi }},\nonumber \\ {\varvec{\Phi }}_t= & {} \left( \begin{array}{cc} {\mathrm {i}\sqrt{6}u}/{2} &{}-\frac{1}{4\lambda }+3u^2+3\sqrt{6}\,\mathrm {i}\, u^2u_{x} \\ \frac{1}{4}+\lambda (-3u^2+3\sqrt{6}\,\mathrm {i}\, u^2u_x)&{} - {\mathrm {i}\sqrt{6}u}/{2} \end{array}\right) \, {\varvec{\Phi }}. \end{aligned}$$

### Equation (4)

The Eq. (4) does not appear to have been considered before in the literature.

*Higher symmetry* The first higher symmetry of the Eq. (4) is

21 $$\begin{aligned} u_{\tau }=\frac{u_{5x}}{(1+6u_{2x})^{\frac{10}{3}}}-30\frac{u_{3x}u_{4x}}{(1+6u_{2x})^{\frac{13}{3}}}+160\frac{u_{3x}^3}{(1+6u_{2x})^{\frac{16}{3}}}. \end{aligned}$$

*Hamiltonian structure and recursion operator* Let $w=(6 u_{2x}+1)^{-\frac{2}{3}}$. Then the symmetry (21) becomes

22 $$\begin{aligned} w_{\tau }=w^5w_{5x}+5 w^4 w_x w_{4x}+\frac{5}{2} w^4 w_{2x}w_{3x}+\frac{15}{4} w^3 w_x^2 w_{3x}=-2 \mathcal{H}\delta _w w^{-1} , \end{aligned}$$

where

$$\begin{aligned} \mathcal{H}=w^{\frac{5}{2}} D_x^2 w^{\frac{3}{2}} D_x w^{\frac{3}{2}} D_x^2 w^{\frac{5}{2}} \end{aligned}$$

is a Hamiltonian operator. In terms of the quantity *w*, its symplectic operator $\mathcal{J}$ has the same form as that for (2), being given by (13). Thus, the recursion operator $\mathcal{R}=\mathcal{H}\mathcal{J}$ generates the symmetries for (4) and

$$\begin{aligned} \mathcal{J}w_{\tau }=\delta _w \left( -w^3 w_{3x}^2+\frac{1}{6} w^2 w_{2x}^3-\frac{1}{4} w w_x^2 w_{2x}^2-\frac{w_x^6}{48 w} \right) . \end{aligned}$$

*Reciprocal transformation* After rescaling *u* and taking $t\rightarrow -t$, the *x* derivative of Eq. (4) can be rewritten in the form

23 $$\begin{aligned} m_t+um_x+\frac{3}{2} u_x m=0, \qquad m= \frac{2}{3}+u_{xx}, \end{aligned}$$

which is a degenerate form of the *b*-family of peakon equations (9), with $b=3/2$. The quantity $m^{2/3}$ is a conserved density, and the conservation law

$$\begin{aligned} p_t+(pu)_x=0, \qquad p=m^{2/3} \end{aligned}$$

can be used to define the reciprocal transformation

$$\begin{aligned} \mathrm {d}X=p \, \mathrm {d}x-pu\, \mathrm {d}t, \qquad \mathrm {d}T= \mathrm {d}t. \end{aligned}$$

Hence, (23) leads to the equations

$$\begin{aligned} \left( p^{-1}\right) _T = u_X, \qquad p^{3/2}=\frac{2}{3}+p\Big (p u_X\Big )_X = \frac{2}{3}+p\left( p\left( p^{-1}\right) _T\right) _X, \end{aligned}$$

and the latter can be rewritten as

24 $$\begin{aligned} (\log p)_{XT}+p^{1/2}-\frac{2}{3}p^{-1}=0, \end{aligned}$$

which is equivalent to the Tzitzeica equation.

*Lax pair* Starting from a $3\times 3$ Lax representation for the Tzitzeica equation, it is straightforward to obtain the following Lax representation for (23):

25 $$\begin{aligned} {\varvec{\Phi }}_x=\left( \begin{array}{ccc} 0 &{} \mathrm {i}\lambda m &{} 0 \\ 0 &{} 0 &{} \mathrm {i}\lambda m \\ -\frac{2}{3}\lambda &{} 0 &{} 0 \end{array}\right) \, {\varvec{\Phi }}, \qquad {\varvec{\Phi }}_t=\left( \begin{array}{ccc} -\frac{1}{2}u_x &{} - \mathrm {i}\lambda um &{} \frac{1}{2}{\lambda }^{-1} \\ \frac{\mathrm {i}}{2}{\lambda }^{-1} &{} 0 &{} - \mathrm {i}\lambda um \\ \frac{2}{3}{\lambda }u &{} \frac{\mathrm {i}}{2}{\lambda }^{-1} &{} \frac{1}{2}u_x \end{array}\right) \, {\varvec{\Phi }}. \end{aligned}$$

### Equation (5)

To the best of our knowledge, the Eq. (5) has not been studied before.

*Higher symmetry* The first higher symmetry of the Eq. (5) is

$$\begin{aligned} u_{\tau }=\left( \frac{u_{4x}}{(1-2u_x)^{\frac{10}{3}}(1+4u_x)^{\frac{5}{3}}} +10\frac{(8u_x-1)u_{2x}u_{3x}}{(1-2u_x)^{\frac{13}{3}}(1+4u_x)^{\frac{8}{3}}}+40\frac{(1-6u_x+24u_x^2)u_{2x}^3}{(1-2u_x)^{\frac{16}{3}}(1+4u_x)^{\frac{11}{3}}}\right) _x . \end{aligned}$$

*Hamiltonian structure and recursion operator* The above symmetry takes the form

$$\begin{aligned} u_{\tau }=\frac{1}{8}\mathcal{H}\delta _u p, \qquad {\mathrm {where}} \quad \mathcal{H}=D_x (1-2 u_x)^{-1}D_x (1-2 u_x)^{-1} D_x \end{aligned}$$

is a Hamiltonian operator, and *p* is given by

26 $$\begin{aligned} p=(1-2u_x)^{2/3}(1+4u_x)^{1/3} . \end{aligned}$$

The symmetry hierarchy of (5) can be generated by the recursion operator $\mathcal{R}=\mathcal{H}\mathcal{J}$, where $\mathcal{J}$ is a symplectic operator given by

$$\begin{aligned} \mathcal{J}=D_x \left( fD_x+D_x f+gD_x^{-1}h+hD_x^{-1}g\right) D_x, \end{aligned}$$

with

$$\begin{aligned}&f=\frac{1}{2(1-2u_x)^{2}(1+4 u_x)^{2}}; \qquad g=\frac{8 u_x}{(1-2u_x)^{\frac{1}{3}}(1+4 u_x)^{\frac{2}{3}}};\\&h=\frac{u_{3x}}{(1-2u_x)^{\frac{8}{3}}(1+4 u_x)^{\frac{7}{3}}}+\frac{2(10u_x-1)}{(1-2u_x)^{\frac{11}{3}}(1+4 u_x)^{\frac{10}{3}}}. \end{aligned}$$

Indeed, we have

$$\begin{aligned} \mathcal{J}u_{\tau }= & {} \delta _u \frac{1}{(4u_x+1)^{11/3}(1-2u_x)^{16/3}}\left( \frac{u_{4x}^2}{2}+\frac{2}{3} \frac{ (60 u_x-7)u_{3x}^2}{(4u_x+1)(2u_x-1)}\right. \\&-\,8 \frac{(360 u_x^2-62 u_x+17) u_{2x}^2 u_{3x}^2}{(4u_x+1)^2(2u_x-1)^2}\\&+\left. \frac{704}{15} \frac{(12960 u_x^4-4032 u_x^3+2340 u_x^2-324 u_x+31) u_{2x}^6}{(4u_x+1)^4(2u_x-1)^4}\right) . \end{aligned}$$

*Reciprocal transformation* The quantity *p* in (26) is a conserved density for (5), with the conservation law

27 $$\begin{aligned} p_t = -4(u^2 p)_x, \end{aligned}$$

leading to the reciprocal transformation

28 $$\begin{aligned} \mathrm {d}X=p\, \mathrm {d}x -4 u^2 p\, \mathrm {d}t, \qquad \mathrm {d}T = \mathrm {d}t. \end{aligned}$$

Under the latter change of independent variables, the conservation law (27) is transformed to the system

29 $$\begin{aligned} \left( p^{-1}\right) _T=4 (u^2)_X, \qquad \frac{(1-2W)^2(1+4W)}{p^3}=1, \quad W=pu_X , \end{aligned}$$

while the Eq. (5) becomes

30 $$\begin{aligned} W_T=\frac{up^2}{\psi }, \end{aligned}$$

where from the second equation in (29), it is consistent to introduce the quantity $\psi$ such that

31 $$\begin{aligned} \psi =\frac{1-2W}{p}, \qquad \frac{1}{\psi ^2}=\frac{1+4W}{p}. \end{aligned}$$

Then by (29) and (30) it follows that

$$\begin{aligned} (\log \psi )_T=-2u, \end{aligned}$$

and by taking the *X* derivative of the latter, using $u_X=W/p$, and taking the difference of the two equations in (31), an equation for $\psi$ alone results, namely

32 $$\begin{aligned} (\log \psi )_{XT}=\frac{1}{3}\Big (\psi -\psi ^{-2}\Big ), \end{aligned}$$

which is a form of the Tzitzeica equation.

*Lax pair* The Eq. (5) has the $3\times 3$ Lax representation

33 $$\begin{aligned} {\varvec{\Phi }}_x= & {} \left( \begin{array}{ccc} 0 &{} 1-2u_x &{} 0 \\ 0 &{} 0 &{} 1+4u_x \\ \lambda (1-2u_x) &{} 0 &{} 0 \end{array}\right) \, {\varvec{\Phi }},\nonumber \\ {\varvec{\Phi }}_t= & {} \left( \begin{array}{ccc} 0&{} - 4u^2(1-2u_x)&{} \frac{1}{3}{\lambda }^{-1} \\ \frac{1}{3}&{} 2u &{} - 4u^2(1+4u_x) \\ -4{\lambda }u^2(1-2u_x) &{} \frac{1}{3} &{} -2u \end{array}\right) \, {\varvec{\Phi }}. \end{aligned}$$

It is interesting to apply the reciprocal transformation (28) to the Lax pair. Upon making this change of independent variables, (33) becomes

34 $$\begin{aligned} {\varvec{\Phi }}_X=\left( \begin{array}{ccc} 0 &{} p^{-1}-2u_X &{} 0 \\ 0 &{} 0 &{} p^{-1}+4u_X \\ \lambda \, (p^{-1}-2u_X) &{} 0 &{} 0 \end{array}\right) \, {\varvec{\Phi }}, \qquad {\varvec{\Phi }}_T=\left( \begin{array}{ccc} 0 &{} 0 &{} \frac{1}{3}\lambda ^{-1} \\ \frac{1}{3} &{} 2u &{} 0 \\ 0 &{} \frac{1}{3} &{} -2u \end{array}\right) \, {\varvec{\Phi }}.\nonumber \\ \end{aligned}$$

The compatibility of the linear system (34) gives

35 $$\begin{aligned} \left( p^{-1}\right) _T=4 (u^2)_X, \qquad u_{XT}-\frac{u}{p}-\left( u^2\right) _X=0 , \end{aligned}$$

where the second equation above arises as a differential consequence of the system (29), and can be integrated to yield the more general equation

$$\begin{aligned} \frac{(1-2W)^2(1+4W)}{p^3} =1+F(X), \qquad W=pu_X , \end{aligned}$$

where *F* is an arbitrary function. However, upon making a point transformation in *X*, so that

$$\begin{aligned} \hat{X}=G(X), \qquad u(X,T)=\hat{u}(\hat{X},T), \qquad p(X,T)= G'(X)^{-1}\hat{p}(\hat{X},T), \end{aligned}$$

the function *F* can be removed by choosing $G(X) =\int (1+F(X))^{1/3}\,\mathrm {d}X$.

The system (29) corresponds to a negative flow in the Sawada-Kotera hierarchy. To see this, it is convenient to use the quantity $\psi$, as defined in (31), and then $\phi$, the first component of the vector ${\varvec{\Phi }}$, satisfies the scalar Lax pair

36 $$\begin{aligned} \phi _{XXX}+V\,\phi _X=\lambda \, \phi , \qquad \phi _T=\frac{1}{3}\lambda ^{-1}\Big (\psi \,\phi _{XX}-\psi _X\,\phi _X\Big ), \end{aligned}$$

where

$$\begin{aligned} V=-\frac{\psi _{XX}}{\psi }. \end{aligned}$$

If *V* is not specified a priori, then the compatibility conditions for the scalar linear system are

37 $$\begin{aligned} V_T =-\psi _X, \qquad \psi _{XX}+V\,\psi =k, \qquad k_X=0, \end{aligned}$$

and, up sending to $T\rightarrow -T$, (36) is equivalent to the Lax pair found for the reciprocally transformed Vakhnenko equation in [32] (see [18] for more details). In the case at hand, we have $k=0$, and substituting for *V* in terms of $\psi$ in the first equation of (37) and integrating produces

$$\begin{aligned} \psi ^2\left( (\log \psi )_{XT}-\frac{\psi }{3}\right) =\frac{\tilde{F}(T)}{3}, \end{aligned}$$

where $\tilde{F}$ is an arbitrary function; after sending $\psi \rightarrow \tilde{F}(T)^{1/3}\,\psi$ and making a point transformation in *T*, this becomes the Tzitzeica equation in the form (32).

### The Hunter–Saxton equation

In addition to the short-wave limit which takes the Camassa–Holm equation [i.e., (9) with $b=2$] to the Eq. (6), a further limit can be applied to remove the linear dispersion term. Taking the limit

$$\begin{aligned} x\rightarrow \epsilon x, \qquad t\rightarrow - \epsilon t,\qquad u\rightarrow \frac{1}{2} u, \qquad \epsilon \rightarrow 0 \end{aligned}$$

produces the equation

38 $$\begin{aligned} (u_t+uu_x)_x=\frac{1}{2}u_x^2; \end{aligned}$$

a similar limit can be applied to remove the linear dispersion from other equations of the form (1). The Eq. (38) was derived by Hunter and Saxton as an asymptotic model of liquid crystals [17]. The *x* derivative of the Hunter–Saxton equation corresponds to geodesic flow on an infinite-dimensional homogeneous space with constant positive curvature (see [21] and references).

*Higher symmetry* The first higher symmetry of the Eq. (6) is

$$\begin{aligned} u_{\tau }=\frac{u_{3x}}{(1+4u_{2x})^{\frac{3}{2}}}. \end{aligned}$$

*Hamiltonian structure and recursion operator* Notice that

$$\begin{aligned} D_x u_{\tau }=-\frac{1}{4} \delta _u \sqrt{1+4 u_{2x}}. \end{aligned}$$

Thus, $D_x$ is a symplectic operator. The symmetries of (6) can be generated by a recursion operator

39 $$\begin{aligned} \mathcal{R}=\mathcal{H}D_x=\left( \frac{1}{1+4 u_{2x}}D_x+D_x \frac{1}{1+4 u_{2x}}-8 u_{\tau } D_x^{-1} u_{\tau }\right) D_x . \end{aligned}$$

The operators $\mathcal{H}$ and $D_x^{-1}$ form a compatible Hamiltonian pair, which is a particular case of case V in Theorem 4 in [33].

*Reciprocal transformation* Considered as a short-wave limit of the Camassa–Holm equation, the *x* derivative of (6) can be written in the form

$$\begin{aligned} m_t =2um_x+4u_xm, \qquad m=1+4u_{xx} , \end{aligned}$$

giving the conservation law

40 $$\begin{aligned} p_t=2\, (up)_x, \qquad p^2=m. \end{aligned}$$

Then introducing *X*, *T* according to

$$\begin{aligned} \mathrm {d}X = p\, \mathrm {d}x + 2up\, \mathrm {d}t , \qquad \mathrm {d}T = \mathrm {d}t, \end{aligned}$$

and setting $W=u_x$, leads to the three equations

41 $$\begin{aligned} W_T=u+W^2, \qquad \left( p^{-1}\right) _T +2u_X=0, \qquad p^2=1+4pW_X, \end{aligned}$$

where the first equation, for $W_T$, comes from (6), the second equation from (40), and the third from the definition of *p* in terms of *m*. Now from the second equation in (41) and the definition of *W* it follows that $(\log p)_T = 2pu_X=2W$, so that upon differentiating the latter with respect to *X* and using the third equation to eliminate $W_X$, an equation for *p* alone results, namely

$$\begin{aligned} (\log p)_{XT}=\frac{1}{2}\Big ( p -p^{-1}\Big ). \end{aligned}$$

Thus, by setting $p=e^{\mathrm {i}\varphi }$, this yields the sine-Gordon equation in the form $\varphi _{XT}=\sin \varphi$.

*Lax pair* A Lax pair for the Hunter–Saxton equation in the form (38) was found in [20]. For Eq. (6), with the inclusion of linear dispersion, a Lax representation is

42 $$\begin{aligned} {\varvec{\Phi }}_x= & {} \left( \begin{array}{cc} 0 &{} 1+4u_{xx} \\ -\lambda &{} 0 \end{array}\right) \, {\varvec{\Phi }},\nonumber \\ {\varvec{\Phi }}_t= & {} \left( \begin{array}{cc} u_x &{}-\frac{1}{4\lambda }+2u+8uu_{xx} \\ \frac{1}{4}-2\lambda u &{} -u_x \end{array}\right) \, {\varvec{\Phi }}. \end{aligned}$$

### The single-cycle pulse equation 

The Eq. (7) was obtained recently by Sakovich [28] as a reduction of a coupled integrable short pulse system due to Feng [11]. Sakovich showed that the envelope soliton solution of (7) can only be as short as one cycle of its carrier frequency, and hence called it the single-cycle pulse equation.

*Higher symmetry* The first higher symmetry of the Eq. (7) is

$$\begin{aligned} u_{\tau }=\frac{u_{3x}}{\left( 1+u_x^2\right) ^3}-3\frac{u_xu_{2x}^2}{\left( 1+u_x^2\right) ^4}. \end{aligned}$$

*Hamiltonian structure and recursion operator* Notice that

$$\begin{aligned} D_x u_{\tau }=\frac{1}{2} \delta _u \frac{u_{2x}^2}{\left( 1+ u_{x}^2\right) ^3}. \end{aligned}$$

Thus $D_x$ is a symplectic operator. The symmetries of (7) can be generated by a recursion operator

$$\begin{aligned} \mathcal{R}=\mathcal{H}D_x=\left( \frac{1}{\left( 1+ u_{x}^2\right) ^2}D_x+D_x \frac{1}{\left( 1+ u_{x}^2\right) ^2}-4 u_xD_x^{-1}u_{\tau }-4 u_{\tau } D_x^{-1} u_x\right) D_x . \end{aligned}$$

The operators $\mathcal{H}$ and $D_x^{-1}$ form a compatible Hamiltonian pair, which is a particular case of case IV in Theorem 4 in [33].

*Reciprocal transformation* From the conservation law

$$\begin{aligned} p_t=\left( u^2\, p\right) _x, \qquad p =1+u_x^2, \end{aligned}$$

the reciprocal transformation

43 $$\begin{aligned} \mathrm {d}X = p\, \mathrm {d}x + u^2 p \, \mathrm {d}t, \qquad \mathrm {d}T = \mathrm {d}t \end{aligned}$$

yields the equations

44 $$\begin{aligned} \left( p^{-1}\right) _T+\left( u^2\right) _X=0, \qquad p=1+p^2u_X^2. \end{aligned}$$

To see how the latter system is related to the sine-Gordon equation, it is most convenient to consider the Lax pair

45 $$\begin{aligned} {\varvec{\Phi }}_X=\left( \begin{array}{cc} -\mathrm {i}A &{} 1 \\ -\lambda &{} \mathrm {i}A \end{array}\right) \, {\varvec{\Phi }}, \qquad {\varvec{\Phi }}_T=\left( \begin{array}{cc} 0 &{} B\,{\lambda }^{-1} \\ -\overline{B} &{} 0 \end{array}\right) \, {\varvec{\Phi }} \end{aligned}$$

where

$$\begin{aligned} A = u_{XX}+p^{-1}p_Xu_X, \quad B = \frac{1}{4}\left( \frac{pu_X+\mathrm {i}}{pu_X-\mathrm {i}}\right) , \quad \overline{B} = \frac{1}{4}\left( \frac{pu_X-\mathrm {i}}{pu_X+\mathrm {i}}\right) . \end{aligned}$$

The compatibility conditions for this Lax pair mean that it is consistent to set

$$\begin{aligned} A=-\frac{1}{2}\vartheta _X, \quad B=\frac{1}{4}e^{\mathrm {i}\vartheta }, \quad \overline{B}=\frac{1}{4}e^{-\mathrm {i}\vartheta }, \end{aligned}$$

where $$\vartheta $$ satisfies

$$\begin{aligned} \vartheta _{XT} +\sin \vartheta =0. \end{aligned}$$

The solution of (44) is given in terms of the variable $\vartheta$ by

$$\begin{aligned} u=-\frac{1}{2}\vartheta _T, \qquad p={\mathrm {sec}}^2(\vartheta /2). \end{aligned}$$

*Lax pair* Using the inverse of the reciprocal transformation (43) to rewrite the Lax pair (45) in terms of the original independent variables *x*, *t* gives a Lax representation for Eq. (7), namely

46 $$\begin{aligned} {\varvec{\Phi }}_x= & {} \left( \begin{array}{cc} -\frac{\mathrm {i}u_{xx}}{1+u_x^2} &{} 1+u_x^2 \\ -\lambda (1+u_x^2) &{} \frac{\mathrm {i}u_{xx}}{1+u_x^2} \end{array}\right) \, {\varvec{\Phi }},\nonumber \\ {\varvec{\Phi }}_t= & {} \left( \begin{array}{cc} -\frac{\mathrm {i}u^2u_{xx}}{1+u_x^2} &{} \frac{(u_x+\mathrm {i})}{4\lambda (u_x-\mathrm {i})} +u^2(1+u_x^2) \\ - \frac{(u_x-\mathrm {i})}{4 (u_x+\mathrm {i})} -{\lambda }u^2(1+u_x^2) &{} \frac{\mathrm {i}u^2 u_{xx}}{1+u_x^2} \end{array}\right) \, {\varvec{\Phi }}. \end{aligned}$$

### Equation (8)

As noted in the remark above, the Eq. (8) combines the nonlinear terms from (6) and (7), but cannot be directly reduced to either equation.

*Higher symmetry* The first higher symmetry of the Eq. (8) is

$$\begin{aligned} u_{\tau }=\frac{u_{3x}+3\frac{\beta }{\alpha }\left( 1+\beta u_x^2\right) \, u_xu_{2x}+\frac{\beta }{2\alpha ^2}\left( 1+\beta u_x^2\right) ^3\, u_x}{\left( \left( 1+\beta u_x^2\right) ^2+4\alpha u_{2x}\right) ^{\frac{3}{2}}} . \end{aligned}$$

This reduces to the first higher symmetry of (6) when $\beta =0$ and $\alpha =1$, but does not behave well in the limit ${\alpha }\rightarrow 0$.

*Hamiltonian structure and recursion operator* Similarly, to the previous case, we have

$$\begin{aligned} D_x u_{\tau }=-\frac{1}{4\alpha ^2} \delta _u\left( \left( 1+\beta u_x^2\right) ^2+4 \alpha u_{2x}\right) ^{\frac{1}{2}}. \end{aligned}$$

A recursion operator is given by

$$\begin{aligned} \mathcal{R}= & {} \left( \frac{1}{\left( 1+\beta u_x^2\right) ^2+4 \alpha u_{2x}}D_x+D_x \frac{1}{\left( 1+\beta u_x^2\right) ^2+4 \alpha u_{2x}}\right. \\&\left. -8 \alpha ^2 u_{\tau } D_x^{-1}u_{\tau }+\frac{2\beta ^2}{\alpha ^2} u_{x} D_x^{-1} u_x\right) D_x . \end{aligned}$$

Notice that when $\beta =0$ and $\alpha =1$, it leads to the recursion operator (39).

*Reciprocal transformation* In this subsection, we will make use of the higher symmetry above to show that the Eq. (8) has a reciprocal link to an equation of third order, given by (56) below, which is a symmetry of the Calogero–Degasperis–Fokas equation. For our purposes, it will be necessary to consider a solution $u=u(x,t,\tau )$, which depends on the time $\tau$ of the higher flow, in addition to *x*, *t*. Our ultimate goal will be to show that, by means of a further change of dependent and independent variables, the third-order equation we obtain can itself be reduced to the sine-Gordon equation.

The Eq. (8) can be rewritten as

47 $$\begin{aligned} u_{xt}=u+vu_{xx}+\frac{1}{2}v_xu_{x}, \qquad v=2{\alpha }u+{\beta }u^2, \end{aligned}$$

which leads to an equation with a form analogous to the Camassa–Holm equation [3], namely

$$\begin{aligned} m_t = vm_x + 2v_x m, \end{aligned}$$

where

48 $$\begin{aligned} m = \left( 1+{\beta }u_x^2\right) ^2 +4{\alpha }u_{xx}, \end{aligned}$$

and this gives the conservation law

49 $$\begin{aligned} p_t=(vp)_x, \qquad p= m^{1/2}. \end{aligned}$$

Upon introducing the reciprocal transformation

$$\begin{aligned} \mathrm {d}X = p\, \mathrm {d}x + pv\, \mathrm {d}t, \qquad \mathrm {d}T = \mathrm {d}t, \end{aligned}$$

the above relations between *u* and *p* are transformed to

50 $$\begin{aligned} \left( p^{-1}\right) _T + 2\left( {\alpha }+ {\beta }u\right) u_X=0, \qquad p^2 = \left( 1+{\beta }p^2u_X^2\right) ^2+4{\alpha }p(pu_X)_X. \end{aligned}$$

In order to identify this symmetry in terms of known integrable equations of third order, we take $u=u(x,t,\tau )$ and extend the above reciprocal transformation to include this additional flow. First of all, note that

$$\begin{aligned} u_\tau = \frac{1}{2{\alpha }}\left( \frac{p_x}{p^2}+\frac{{\beta }u_x{P}}{{\alpha }p}\right) , \qquad {P}=1+{\beta }u_x^2, \end{aligned}$$

which allows us to write

$$\begin{aligned} \mathrm {d}X = p\, \mathrm {d}x + pv\, \mathrm {d}t +F\, \mathrm {d}\tau , \qquad \mathrm {d}T = \mathrm {d}t, \qquad \mathrm {d}S=\mathrm {d}\tau , \end{aligned}$$

where

$$\begin{aligned} F=\frac{p_{xx}}{p^3}-\frac{3p_x^2}{2p^4}+\frac{{\beta }{P}}{4{\alpha }^2}\Big (3-\frac{{P}^2}{p^2}\Big ). \end{aligned}$$

By applying the above reciprocal transformation to the symmetry, we consider the ratio $\rho = p/{P}$, and then set

$$\begin{aligned} \rho = \frac{p}{{P}}=e^{\mathrm {i}\vartheta } \end{aligned}$$

to find the equation

51 $$\begin{aligned} \vartheta _S=\vartheta _{XXX}+\frac{1}{2}\vartheta _X^3 -\frac{3{\beta }}{2{\alpha }^2}\,\vartheta _X\sin ^2 \vartheta , \end{aligned}$$

which is a form of the Calogero–Degasperis–Fokas equation (see [4, 14, 16]). The Eq. (51) is related via the Miura transformation

52 $$\begin{aligned} y=-\mathrm {i}\, \frac{\rho _X}{\rho } -\frac{\sqrt{{\beta }}}{2{\alpha }}\Big ( \rho - \rho ^{-1}\Big ) =\vartheta _X-\mathrm {i}\frac{\sqrt{{\beta }}}{{\alpha }}\sin \vartheta \end{aligned}$$

to the modified KdV (mKdV) equation in the form

53 $$\begin{aligned} y_S=y_{XXX}+\frac{3}{2} y^2y_X. \end{aligned}$$

Thus we see that under a reciprocal transformation, the Eq. (8) corresponds to a symmetry of the mKdV equation.

The calculations involving the reciprocal transformation are most conveniently carried out by introducing the variable $W=u_x$, so that (8) becomes

$$\begin{aligned} W_T =u {P}+{\alpha }W^2, \qquad {\mathrm {with}}\quad W=pu_X, \quad {P}=1+{\beta }W^2, \end{aligned}$$

and the second equation in (50) gives

54 $$\begin{aligned} W_X = \frac{p^2-{P}^2}{4{\alpha }p} \implies \frac{W_X}{{P}}=\frac{\mathrm {i}}{2{\alpha }}\, \sin \vartheta . \end{aligned}$$

Then, for $\rho = p/{P}$, we find

55 $$\begin{aligned} (\log \rho )_T=\frac{2{\alpha }W}{{P}} \implies \frac{W}{{P}}=\frac{\mathrm {i}}{2{\alpha }}\, \vartheta _T. \end{aligned}$$

Upon computing the *X* derivative of both sides, this yields

$$\begin{aligned} (\log \rho )_{XT}+\frac{1}{2}\Big ( \rho - \rho ^{-1}\Big ) = \frac{1}{{P}}\Big ( \rho - \rho ^{-1}\Big ) \end{aligned}$$

or equivalently

$$\begin{aligned} \frac{2}{{P}} = \frac{\vartheta _{XT}}{\sin \vartheta }+1, \end{aligned}$$

which indicates that the symmetry of the Calogero–Degasperis–Fokas equation corresponding to (8) is not the sine-Gordon equation, but something of higher order. Indeed, differentiating the above equation with respect to *X* and using ${P}_X=2{\beta }WW_X$ together with (54) and (55) leads to the third-order equation

56 $$\begin{aligned} \left( \frac{\vartheta _{XT}}{\sin \vartheta }\right) _X+ \frac{{\beta }}{{\alpha }^2}\Big (\cos \vartheta \Big )_T =0. \end{aligned}$$

In terms of these transformed coordinates, the Lax pair for the *T* flow takes the form

57 $$\begin{aligned} {\varvec{\Phi }}_X=\left( \begin{array}{cc} \frac{\mathrm {i}y}{2} &{} 1 \\ -\lambda &{} -\frac{\mathrm {i}y}{2} \end{array}\right) \, {\varvec{\Phi }}, \qquad {\varvec{\Phi }}_T=\left( \begin{array}{cc} 0 &{} \eta \,{\lambda }^{-1} \\ \zeta &{} 0 \end{array}\right) \, {\varvec{\Phi }} , \end{aligned}$$

where *y* is given by (52) and

58 $$\begin{aligned} \eta =-\frac{e^{\mathrm {i}\theta }}{4}\left( \frac{\theta _{XT}}{\sin \theta } +\frac{\sqrt{{\beta }}}{2{\alpha }} \theta _T \right) , \qquad \zeta = -\frac{\mathrm {i}}{2}y_T-\eta . \end{aligned}$$

With the introduction of the KdV potential

59 $$\begin{aligned} \mathrm {V}= -\frac{\mathrm {i}}{2}y_X+\frac{1}{4}y^2, \end{aligned}$$

this corresponds to the negative KdV flow (see [18]) given by

60 $$\begin{aligned} \mathrm {V}_T=2\eta _X, \qquad \eta _{XXX}+4\mathrm {V}\eta _X + 2\mathrm {V}_X\eta =0, \end{aligned}$$

but not the general solution of this. Indeed, integration of the second equation in (60) gives

$$\begin{aligned} \eta \eta _{XX}-\frac{1}{2}\eta _X^2+2\mathrm {V}\eta ^2 = F(T), \end{aligned}$$

[a form of the Ermakov–Pinney equation, cf. equation (4) in [5]] but from the expression (58) and (56) it follows that

$$\begin{aligned} F(T)=0, \qquad y = -\mathrm {i}(\log \eta )_X, \qquad \mathrm {V}=-\frac{\eta _{XX}}{2\eta }+\frac{\eta _X^2}{4\eta ^2}, \end{aligned}$$

in accordance with the compatibility of the linear system (57). Substituting the latter expression for $\mathrm {V}$ in terms of $\eta$ into the first equation in (60) yields an equation of third order for $\eta$, which integrates to yield

$$\begin{aligned} \eta (\log \eta )_{XT}+2\eta ^2 = 2G(T), \end{aligned}$$

and, after rescaling $\eta \rightarrow \sqrt{G(T)}\, \eta$ and redefining *T* so that $\partial _T \rightarrow \sqrt{G(T)}\, \partial _T$, we see that $\varphi =\mathrm {i}\log \eta$ satisfies the sine-Gordon equation in the form $\varphi _{XT}+4\sin \varphi = 0$.

*Lax pair * In order to obtain a Lax pair for the Eq. (8), it is sufficient to rewrite (57) in terms of the original independent variables *x*, *t*. However, due to the dependence on *p*, this does not directly produce matrices which are rational functions of the original field *u* and its derivatives. To obtain a rational Lax pair, it is convenient to put (57) into scalar form and carry out a gauge transformation, which leads to the scalar linear system

61 $$\begin{aligned}&\psi _{xx}+\left( m{\lambda }+ \frac{f_{xx}}{f}-2\, \frac{f_x^2}{f^2}\right) \, \psi =0, \nonumber \\&\psi _t = \left( v - \frac{1}{4f^2{\lambda }}\right) \psi _x -\left( \frac{1}{2}v_x+\frac{f_x}{4f^3{\lambda }}\right) \,\psi , \end{aligned}$$

where *m* is given in (48), *v* is as in (47), and

$$\begin{aligned} f=1+\mathrm {i}\sqrt{{\beta }}\, u_x. \end{aligned}$$

The scalar system (61) can readily be put into matrix form if desired.

## Conclusions

The list of integrable generalized short pulse equations appears to contain three new equations, namely (4), (5), and also (8), which combines the nonlinear terms of the Hunter–Saxton equation and the single-cycle pulse equation. All of the equations considered here are related by a reciprocal transformation to either the sine-Gordon equation or the Tzitzeica equation. However, although Eqs. (2), (4) and (5) are all related to the Tzitzeica equation, by comparing the expression for the differential $\mathrm {d}X=p\, \mathrm {d}x+\cdots$ in each case, it is apparent that there are no direct links of Bäcklund type between these three equations, without changing the independent variable *x* via a hodograph-type transformation; the same remark applies to the Eqs. (3), (6) and (7). In the case of Eq. (8), the link to the sine-Gordon equation is rather indirect, and the equation that arises directly is the symmetry (56) of the Calogero–Degasperis–Fokas equation, which does not seem to have been considered before. These reciprocal links should be examined further, in order to derive explicit solutions of the new equations in parametric form. Since the reciprocal transformation is only defined for sufficiently smooth solutions, it is worth investigating situations where it breaks down: these equations may admit interesting weak solutions, e.g., distributions with non-empty singular support, as is the case for the *b*-family (9) mentioned above.
